# The Paradox of Life Extension with Traditional Cost-Effectiveness Analysis: A Case Study with Duchenne Muscular Dystrophy

**DOI:** 10.36469/001c.156118

**Published:** 2026-02-18

**Authors:** Lauren Sedita, Alexa Klimchak, Eleanor Perfetto, Katherine Gooch, Daniel C. Malone

**Affiliations:** 1 Sarepta Therapeutics, Cambridge, Massachusetts; 2 University of Maryland, Baltimore; 3 College of Pharmacy, University of Utah, Salt Lake City

**Keywords:** cost-effectiveness analysis, Duchenne muscular dystrophy, progression, quality-adjusted life-years, utility

## Abstract

**Background:**

Traditional quality-adjusted life-year (QALY)–based cost-effectiveness analyses (CEAs) may inadvertently penalize treatments extending survival in populations with disabilities. Duchenne muscular dystrophy (DMD) is a rare, progressive disease diagnosed during childhood with significant morbidity, premature mortality, and considerable healthcare costs. The impact of delaying mortality on CEAs for DMD is unclear.

**Objective:**

To evaluate the impact of delaying nonfatal progression and/or mortality using a QALY-based CEA for three hypothetical DMD treatments, assessing if treatment value increases by delaying morbidity and/or mortality.

**Methods:**

A previously published, five-state QALY-based cost-effectiveness model for analyzing treatments in an early ambulatory DMD population was replicated, validated, and adapted to include an early nonambulatory (ENA) population. Maximum annual treatment price was determined for three hypothetical treatments individually compared for each population with standard of care (SoC): (A) delays nonfatal progression and mortality; (B) delays nonfatal progression; or (C) delays mortality. A 10-year delay was assessed for all three. Willingness-to-pay (WTP) thresholds ranged from 50 000to200 000/QALY.

**Results:**

In both populations, QALYs gained vs SoC were highest for the hypothetical treatment delaying both nonfatal progression and mortality. Maximum annual treatment price was highest for the hypothetical treatment delaying nonfatal progression only (both populations and all WTP thresholds). Delaying mortality alone consistently had a negative annual valuation (not cost-effective even at 0price),rangingfrom−8685 to −2148(EApopulation)and−13 253 to $−3278 (ENA population). For all treatments, valuation for the ENA population was consistently lower.

**Discussion:**

These model results illustrate that delaying both nonfatal progression and mortality is less valuable in a traditional CEA than delaying nonfatal progression alone, even when the additional survival is at the patient’s best possible health and lowest costs. These results emphasize the significant shortcomings of QALY-based CEAs for assessing the value of potential life-extending treatments for progressive, disabling diseases, like DMD.

**Conclusions:**

These results suggest that within a traditional CEA, delaying mortality decreases a DMD treatment’s value estimate, countering society’s values and norms for desired life extension in vulnerable populations. These limitations must be carefully considered when interpreting model results and highlight the need for applying other value assessment methodologies.

## INTRODUCTION

Cost-effectiveness analyses (CEAs) attempt to estimate the relative value of a treatment or intervention by evaluating its costs and benefits compared with an alternative. Health technology assessment bodies use CEA results to determine access and/or inform reimbursement decisions.^1^ In the United States, CEAs are increasingly cited in commercial payer policies^2,3^ and are a tool to inform payer decisions regarding patient access to treatment. It is imperative, therefore, that CEAs equitably estimate the valuation of all treatments while limiting biases that could result from unique attributes of specific diseases and/or treatments. It is also important that CEAs reflect the clinical, economic, and societal benefits of treatment. However, most take only the perspective of the payer or healthcare system.

CEAs commonly use quality-adjusted life-years (QALYs) to quantify the health benefits derived from treatments. As a single metric, QALYs are calculated by incorporating the amount of time a patient spends in a given stage of a disease. That time is weighted by the estimated health-related quality of life (HRQoL) in that state, which is known as the health state utility. However, considerable concerns have arisen regarding the use of the QALY and the estimations of HRQoL. These concerns often focus on the application of the QALY in evaluating treatments that extend survival,^4–7^ particularly in populations with low baseline utilities due to disability, severe illness, or advanced age.^8^ Other concerns involve how health state utility is measured, including the large impact of ambulation ability on total scores.^9^

While concerns related to treatments for disabling diseases were largely considered to be theoretical, they have gained more recent attention because advancements in science are resulting in treatments for younger individuals with severe conditions. One such disease is Duchenne muscular dystrophy (DMD), a rare, genetic, progressive disease diagnosed in childhood. DMD is characterized by muscle weakness at an early age that eventually progresses to loss of ambulation, loss of upper body function, pulmonary and cardiac decline, and early mortality.^10,11^ Although timing of disease progression is heterogeneous, patients follow a predictable pattern through key stages of disease progression typically captured in four distinct health states. Younger, early ambulatory (EA) patients, who are often unable to keep up with their peers, usually have fewer or less severe symptoms of physical decline. Patients who progressed from the late ambulatory (LA) to early nonambulatory (ENA) state have lost the ability to walk independently. However, these patients are able to perform many activities of daily living (eg, eating, self-care, writing, use of computers/video games/phones) and generally do attend school due to some maintenance of upper body function.^12^ Patients eventually progress into the late nonambulatory (LNA) state. As the disease progresses, costs generally increase while HRQoL decreases.^13–15^

Using DMD as a case study, the current analyses sought to assess how treatment valuation in DMD is driven by delaying progression and/or delaying mortality in different stages of the disease using a traditional QALY-based value assessment approach.

## METHODS

### Model Structure and Inputs

A five-state, partitioned survival DMD model published by the Institute for Clinical and Economic Review (ICER),^16^ was replicated (ie, same standard of care [SoC] risks, direct medical costs, utilities, and 3% discount rate for costs and benefits) and validated by comparing against published results. As in the ICER report, the following utilities were assigned to each health state: EA = 0.73; LA = 0.64; ENA = 0.21; and LNA = 0.18. The model was adapted for two DMD populations: (1) 5-year-old EA patients (initial health state with higher utility/lower cost) and (2) 13-year-old ENA patients (initial health state with lower utility/higher cost). This study was conducted using a US healthcare payer perspective with a lifetime horizon.

### Treatment Benefits

Three different hypothetical treatments, with varying effects on morbidity and mortality, were evaluated and compared with SoC (**Table 1**). Treatment A would delay nonfatal disease progression and mortality, resulting in a rightward shift of both nonfatal and mortality risk curves such that patients spend more time in their starting health state. In this context, the patient lives longer than if they were treated with SoC, and that additional time is at their highest possible utility value with lowest possible direct medical cost.

**Table 1.** Description of Treatments

**Treatment**	**Description**	**Pause in Nonfatal Progression (Rightward Shift of Nonfatal Risk Curves)**	**Pause in Mortality (Rightward Shift of Mortality Risk Curve)**
**Treatment A**	Disease progression is temporarily halted immediately Patients spend additional 10 years in their starting health state before disease progression resumes at the SoC rate Patients live longer than with SoC alone	Yes	Yes
**Treatment B**	Disease progression is temporarily halted immediately Patients spend additional time in their starting health state but do not live longer compared to SoC alone Some patients may therefore die before progressing through all DMD health states or before the full 10-year pause has been realized	Yes	No
**Treatment C**	Disease progression is temporarily halted once patients reach the most advanced health state (LNA), regardless of their starting health state Patients spend an additional 10 years in the LNA health state and live longer than with SoC alone All life extension happens in LNA	No	Yes

Abbreviations: DMD, Duchenne muscular dystrophy; LNA, late nonambulatory; SoC, standard of care.

Treatment B would delay disease progression only, resulting in a rightward shift of only nonfatal risk curves. Thus, patients spend more time in their starting health state with Treatment B than with SoC but have the same overall survival. In this hypothetical scenario, mortality could precede progression to more severe DMD health states.

Treatment C would delay mortality only, resulting in a rightward shift of the mortality risk curve. In this scenario, patients spend more time in the LNA health state (lowest utility and highest costs) than with SoC, regardless of their starting health state.

Understanding that some aspects of these hypothetical treatments may be clinically unrealistic, the attributes of these hypothetical treatments are required to test the mathematical capabilities of a QALY-based CEA for DMD.

### Base Case Analysis

A 10-year pause in disease progression was assessed in the base case for all three treatments. Although the same pause was used for all treatments, the treatments differed in how the 10-year pause was applied: both the nonfatal risk and mortality risk curves (Treatment A), the nonfatal risk curves only (Treatment B), or the mortality risk curve only (Treatment C). These rightward shifts in the risk curves are aligned with what has been assumed in other cost-effectiveness studies of DMD treatments.^16–18^

The main results produced were the maximum annual treatment price at the following willingness-to-pay (WTP) thresholds, all of which align with commonly employed WTP thresholds^19,20^: $50 000/QALY, $100 000/QALY, $150 000/QALY, and $200 000/QALY. Maximum (cost-effective) treatment price was calculated in lieu of an incremental cost-effectiveness ratio with a hypothetical price.

### Scenario Analyses

Given its increasing use in value assessments as an alternative to the QALY measure,^21^ the first scenario analysis calculated the maximum annual treatment prices based on the equal value of life-years gained (evLYG) metric, with each additional year of survival beyond SoC having a utility value of 0.851 (reflecting the average utility in the general population).^22^

In the second scenario analysis, direct medical costs were removed in instances where the hypothetical treatment was not cost-effective even at a price of zero, which aligns with scenario analyses proposed by both ICER and the National Institute for Health and Care Excellence (NICE).^21,23,24^

In the final scenario analysis, a 5- and 20-year pause was assessed for all three treatments. Given the expected survival of patients with DMD treated with SoC (median life expectancy of 24 years in the ICER model^16^), nothing beyond a 20-year pause was assessed. In addition, the impact of health benefit discounting was assessed with a 1.5% discount rate.

## RESULTS

### Base Case Analysis

In the EA population, the maximum annual treatment price for Treatment A ranged from $7437 to $41 857; for Treatment B, from $18 261 to $51 493; and for Treatment C, from $−8685 to $−2148 (**Table 2**). The valuation of Treatment B was highest per year across all WTP thresholds compared with the other two treatments. Thus, delaying nonfatal progression alone provided the most value for this group of patients. The greatest QALY gains came from Treatment A (4.62). However, Treatment B reduced direct medical costs (lifetime incremental costs were $−109 694) because patients receiving Treatment B were modeled to be in less severe (less costly) health states prior to death. Patients receiving Treatment A were modeled to spend time in all health states, including severe health states associated with higher healthcare costs. Thus, Treatment B had the best valuation. In contrast, Treatment C had the lowest QALY gains (0.88) and the most incremental costs incurred ($218 901), resulting in the lowest valuation. While Treatments A and B both had positive maximum annual treatment prices, the annual threshold price of Treatment C was negative at all WTP thresholds (**Table 2**). Thus, an intervention (Treatment C) with a 10-year delay in mortality only would not be cost-effective even at a $0 price. Markov traces for each of the treatments are shown in **Figure S1**.

**Table 2.** Maximum Annual Treatment Prices Based on QALYs Gained at Various Willingness-to-Pay Thresholds in the EA Population

	**Treatment A**	**Treatment B**	**Treatment C**
QALYs gained vs SoC	4.62	3.38	0.88
Incremental costs vs SoC, $	81 317	-109 694	218 901
Total LYs	20.15	15.27	20.15
Maximum annual treatment price, $			
$50 000/QALY	7437	18 261	-8 685
$100 000/QALY	18 911	29 338	-6506
$150 000/QALY	30 384	40 416	−4327
$200 000/QALY	41 857	51 493	−2148

Abbreviations: EA, early ambulatory; LY, life-year; QALY, quality-adjusted life-year; SoC, standard of care.

Maximum annual treatment price represents the average across the full treatment time, which is different between Treatments A and C (20.15 discounted LY) and Treatment B (15.27 discounted LY). Treatment A delays nonfatal progression and mortality; Treatment B delays nonfatal progression only; Treatment C delays mortality only.

Similar to the EA population, the valuation of Treatment B was highest per year across all WTP thresholds in the ENA population vs the other two treatments (**Table 3**). Therefore, the most value was in delaying nonfatal progression alone, even in patients starting in a more progressed, nonambulatory health state. Although the highest QALY gains came from Treatment A, they were much lower than when Treatment A was given to EA patients (1.32 vs 4.62 QALYs, respectively). Treatment B had the lowest QALYs gained (0.13), but the incremental direct medical cost offsets resulted in a positive valuation across all WTP thresholds. Even though Treatment C no longer had the lowest QALYs gained when given to the ENA cohort, it still had a negative valuation, ranging from $−13 253 to $−3278 per year. Thus, delaying mortality by 10 years would not be cost-effective even when the treatment was assigned a $0 price (**Table 3**). For all treatments, the valuation was higher when given to the EA population compared to the ENA population, despite equal pauses in disease progression.

**Table 3.** Maximum Annual Treatment Prices Based on QALYs Gained at Various Willingness-to-Pay Thresholds in the ENA Population

	**Treatment A**	**Treatment B**	**Treatment C**
QALYs gained vs SoC	1.32	0.13	1.10
Incremental costs vs SoC, $	193 301	−47 347	274 407
Total LYs	16.55	10.44	16.55
Maximum annual treatment price, $			
$50 000/QALY	−7699	5142	−13 253
$100 000/QALY	−3719	5748	−9928
$150 000/QALY	260	6354	−6603
$200 000/QALY	4240	6960	−3278

Abbreviations: ENA, early nonambulatory; LY, life-year; QALY, quality-adjusted life-year; SoC, standard of care.

Maximum annual treatment price represents the average across the full treatment time, which is different between Treatments A and C (16.55 discounted LY) and Treatment B (10.44 discounted LY). Treatment A delays nonfatal progression and mortality; Treatment B delays nonfatal progression only; Treatment C delays mortality only.

### Scenario Analyses

For the analyses conducted using the evLYG metric,^21^ the values for Treatments A and C increased but (by definition) remained the same for Treatment B due to the lack of a modeled mortality benefit (**Tables 4** and **5)**. Treatment A was the most valuable with WTP thresholds of $100 000/QALY or greater for both populations, as it provided the highest value in terms of health benefits. At a WTP of $50 000/QALY, Treatment B remained the most valuable. Treatment C still had negative valuation (not cost-effective at any price) in both populations at this WTP threshold.

**Table 4.** Maximum Annual Treatment Prices Based on evLYG at Various Willingness-to-Pay Thresholds in the EA Population

	**Treatment A**	**Treatment B**	**Treatment C**
evLYG vs SoC	7.53	3.38	4.15
Incremental costs vs SoC, $	81 317	−109 694	218 901
Total LYs	20.15	15.27	20.15
Maximum annual treatment price, $			
$50 000/QALY	14 661	18 261	−563
$100 000/QALY	33 359	29 338	9 739
$150 000/QALY	52 056	40 416	20 041
$200 000/QALY	70 753	51 493	30 343

Abbreviations: EA, early ambulatory; evLYG, equal value of life-years gained; LY, life-year; QALY, quality-adjusted life-year; SoC, standard of care.

Maximum annual treatment price represents the average across the full treatment time, which is different between Treatments A and C (20.15 discounted LY) and Treatment B (15.27 discounted LY). Treatment A delays nonfatal progression and mortality; Treatment B delays nonfatal progression only; Treatment C delays mortality only.

**Table 5.** Maximum Annual Treatment Prices Based on evLYG at Various Willingness-to-Pay Thresholds in the ENA Population

	**Treatment A**	**Treatment B**	**Treatment C**
evLYG vs SoC	5.33	0.13	5.20
Incremental costs vs SoC, $	193 301	−47 347	274 407
Total LYs	16.55	10.44	16.55
Maximum annual treatment price, $			
$50 000/QALY	4424	5142	−858
$100 000/QALY	20 526	5748	14 862
$150 000/QALY	36 628	6354	30 582
$200 000/QALY	52 730	6960	46 302

Abbreviations: ENA, early nonambulatory; evLYG, equal value of life-years gained; LY, life-year; QALY, quality-adjusted life-year; SoC, standard of care.

Maximum annual treatment price represents the average across the full treatment time, which is different between Treatments A and C (16.55 discounted LY) and Treatment B (10.44 discounted LY). Treatment A delays nonfatal progression and mortality; Treatment B delays nonfatal progression only; Treatment C delays mortality only.

In the second scenario analysis, costs for Treatment C were removed as this hypothetical treatment was not cost-effective even at a price of zero in the base case. By valuing Treatment C on health benefits (QALYs) only, the treatment valuation became positive for both populations (**Table S1**). For the EA population, Treatment C valuation was still lower than the valuations for Treatment A and Treatment B in the base case at all WTP thresholds. However, for the ENA population, Treatment C’s value in this scenario was higher than Treatment A in the base case at all WTP thresholds, and Treatment B in the base case at WTP of $100 000/QALY or higher. For both populations, at a WTP of $100 000 or higher, the value was lower than that with the evLYG.

The same trend of Treatment B having the highest valuation of all treatments was observed with the final scenario analyses with a 5-year and 20-year pause. The only exception was for EA patients, with a 20-year pause at a WTP of $200 000/QALY where Treatment A had a higher annual valuation than Treatment B by $102 (**Tables S2-S5**). Decreasing the benefit discount rate to 1.5% increased the valuation of all treatments. However, the results remained largely consistent with the base case in both populations, with the only exception for ENA patients at a WTP of $200 000/QALY with a higher annual valuation for Treatment A vs Treatment B by $399 (**Tables S6** and **S7**). The valuations of treatments were consistently greater for the EA population compared with the ENA population. Treatment C was only cost-effective in the EA and ENA populations at the highest WTP threshold analyzed in this scenario.

## DISCUSSION

The present analysis provides an example of how a hypothetical DMD treatment that extends survival could be penalized within a traditional QALY-based CEA value assessment. Although Treatment A provided the most health benefit in delaying both progression and mortality, Treatment B had the highest valuation for both DMD populations despite only delaying nonfatal disease progression ($18 911 vs $29 339 per year, respectively at the commonly used WTP of $100 000/QALY). Thus, a traditional CEA for DMD conducted from a payer perspective could be interpreted as a treatment having greater value if patients died rather than lived in the LNA health state, which conflicts with societal preferences and norms.^25–28^

It is worth noting that Treatment A added years of life at the patient’s highest possible utility and lowest possible cost, such as when patients in the EA population have milder symptoms or when patients in the ENA population maintain good upper body function, exhibit independence, and can perform many activities of daily living. Nonetheless, Treatment B was estimated to be more valuable than the treatments that delayed mortality because it reduced the time a patient spent in nonambulatory health states relative to SoC alone. Because those health states have low values for utility and high direct medical costs, time spent in those health states will reduce the overall valuation of a therapy. Furthermore, Treatment C, which only delayed mortality and resulted in patients spending more time in the LNA health state, resulted in a negative maximum annual treatment price for both populations. Thus, Treatment C would not be deemed cost-effective even if it were provided at no cost.

The impact of two CEA modifications (ie, use of the evLYG and removal of treatment costs in instances where the treatment was not cost-effective at any price) was examined in separate scenario analyses. At a WTP of $100 000/QALY or higher, Treatment A became the most valuable when measured with the evLYG, which differs from the base case results that utilized the QALY. Use of evLYG may be informative in the evaluation of treatments that extend life, especially in instances where disease progression is irreversible. Furthermore, while removal of treatment costs for Treatment C increased its value above $0, this scenario analysis is recommended only in certain situations, making comparisons of valuations across treatments difficult. This modification would not impact treatments with very low, yet still positive, treatment valuations. Further analyses may include additional potential solutions, such as multicriteria decision analysis or integrating patient-weighted utilities.

The same trends observed in the base case were observed in the final scenario analyses using traditional QALY-based assessments, with two exceptions. Regarding treatments that would result in a 20-year pause with a WTP of $200 000/QALY in the EA patients, the larger rightward shift of risk curves combined with higher WTP might be able to overcome this survival paradox in this population (who have higher baseline utility and lower cost). These results complement a recent study analyzing the valuation of a hypothetical treatment for DMD equivalent to Treatment A, which demonstrated substantially lower valuation when given to an ENA population vs an EA population.^8^ They are also consistent with published concerns that treatments for nonambulatory, chronic conditions may be deemed less cost effective using QALY-based CEAs, leading to bias against certain patient populations.^9^

The other exception was treatments that would result in a 10-year pause with 1.5% benefit discounting and a WTP of $200 000/QALY in the ENA patients. The lower discount rate for benefits combined with higher WTP may also be able to overcome the survival paradox in the ENA population, especially given the relatively small impact of lowering the discount rate on the QALY gains with Treatment B compared with those with Treatment A.

While there are many potential perspectives to consider when determining the value of a treatment, it is important to assess whether the relative valuation reflects society’s norms and preferences and/or the preferences of patients in the target population and their families. Societal norms and preferences for prioritizing healthcare resources are captured in a variety of ways. For example, in embracing the welfare economic premise for conducting CEAs, spending money on medical treatments is not out of line with how society has chosen to spend its resources. In a “fair-innings” argument, society wishes to give everyone an opportunity at a full, healthy life, which may lead to prioritizing treatments for younger patients such that they can achieve a “normal” lifespan.^27,28^ Under the Rule-of-Rescue premise, spending money in rescuing people from avoidable death is foremost despite the relatively small QALYs gained compared with spending that money on other things.^26^ In a discrete choice experiment of the US general population, respondents valued life expectancy and symptom burden as the most important disease attributes for prioritizing research and treatment.^25^ These perspectives suggest that societal values contrast with the findings derived from the QALY-based model studied in this analysis.

In line with societal preferences, patients with DMD and their families place high value on a treatment that can delay both progression and mortality. Patient-preference research has shown that DMD patients and caregivers prioritize cardiac and pulmonary symptoms, which strongly impact lifespan in DMD, as important treatment targets.^29,30^ Chance of heart and lung benefits were considered relatively important in a hypothetical hierarchy exercise regarding clinical trial decision-making.^31^ Another DMD caregiver preference study demonstrated positive utility (via best-worst scaling) for a treatment that offered improvements to life expectancy of 2 or 5 years, vs a negative best-worst score for a treatment that offered no change in life expectancy.^32^ Patients also value treatments that extend survival until a time when new, improved therapies are available (ie, option value).^33,34^ Thus, while extending life and delaying progression are both typically preferred by society, patients, and families, this study demonstrates these preferences may not be represented within traditional QALY-based value assessment methods, given that the core economic logic underpinning of traditional CEA methodology seeks to maximize efficiency (ie, QALYs per dollar). Functionally, this economic logic penalizes each year spent in high-cost/low-utility states (eg, the nonambulatory states), as it results in a negative value, which paradoxically reduces the value assessment of any treatment that can offer survival benefits important to patients and their families.

While this study took a healthcare perspective similar to that of many CEAs, it is possible that a societal perspective could shift the results, potentially leading to a positive valuation for life extension in nonambulatory health states. However, depending on the specific inputs and how certain elements (eg, caregiver burden, work productivity) are incorporated into a model, including a societal perspective could negatively impact treatment value. For example, inclusion of caregiver burden may further emphasize the paradox seen in this study (ie, caregiver trap) as treatments that extend life also extend caregiver responsibilities (contributing to further carer QALY losses) as previously described for DMD^35^ and prolong the decreases in productivity noted with DMD progression.^36^ Therefore, it would be especially important to consider bereavement effects when incorporating broader perspectives.^37,38^ The model used in this study was based on a model in a published ICER DMD value assessment report, which included an analysis from a “modified” societal perspective.^16^ That perspective did not significantly alter the results,^16^ suggesting that inclusion of nondirect medical costs and caregiver utility would not change the conclusions made in this study. In general, because utilities decrease substantially when patients lose ambulation, QALYs will always be lower in DMD nonambulatory states compared with ambulatory ones. Therefore, from any CEA perspective, it is expected that time in the nonambulatory states will have a lower valuation (and may even be negative), assuming the treatment cannot reverse loss of ambulation and costs (direct medical as well as societal) do not decrease with progression.

The present study has limitations that should be kept in mind when interpreting the results. The benefits of the three hypothetical treatments may be clinically unrealistic (eg, delaying nonfatal progression or mortality alone, or increasing survival time in one health state only) and do not reflect the real-world benefits obtained from existing DMD treatments. The purpose of this study was not to compare current treatments but to provide methodological insights on the mechanics of traditional, QALY-based CEAs by assessing the relative treatment valuation of delaying nonfatal progression and/or mortality within this framework. Thus, these assumptions were necessary to avoid confounding factors. This study focused on a specific published CEA and did not assess other inputs that could affect the results of traditional CEAs. Furthermore, the cost-effectiveness model was built from the payer perspective only and did not consider societal or patient perspectives, or other components of value. The study also assumed fixed utility values for health states, which followed the replicated model but may not capture individual patient heterogeneity in quality-of-life preferences. Finally, the analysis only focused on DMD; therefore, additional research is needed to determine if these insights apply to other diseases, including other diseases that are both disabling and life-shortening.

## CONCLUSION

Replicating a traditional CEA framework, this study demonstrated that a hypothetical DMD treatment that could potentially delay mortality was less valuable than one that only delays nonfatal progression, even when the additional years of survival are at the patient’s best possible health and lowest costs. These findings suggest that treatments have greater value if the patient’s lifespan is shortened rather than treatment extending survival in nonambulatory health states with lower utilities and higher direct medical costs. These findings highlight methodological limitations of traditional CEA framework and their apparent conflict with common patient and societal preferences for life extension.^25–28^ These results contribute to the growing evidence that highlights the need to use caution when interpreting results from QALY-based CEAs for disabling diseases like DMD. The findings also underscore the importance of applying other value assessment approaches to avoid any discriminatory implications for disabled populations and developing and testing approaches to overcome these limitations.

### Conflict of Interest

L.E.S., A.C.K., and K.L.G. are employees of Sarepta Therapeutics, Inc. and may own stock/options in the company. D.C.M. and E.M.P. have served as consultants to Sarepta Therapeutics, Inc.

## Supplementary Material

Online Supplementary Material
