# Supplementary material for: The Paradox of Life Extension with Traditional Cost-Effectiveness Analysis: A Case Study with Duchenne Muscular Dystrophy
Source: J Health Econ Outcomes Res. 2026 Feb 18;13(1):56–62. doi: 10.36469/001c.156118 (PMC12922800; doi:10.36469/001c.156118)
Supplement: Online Supplementary Material [file jheor_2026_13_1_156118_332128.pdf]

Journal of Health Economics and Outcomes Research

Online Supplementary Material

The Paradox of Life Extension with Traditional Cost-Effectiveness Analysis: A Case Study with Duchenne Muscular Dystrophy. *JHEOR*. 2026;13(1):56-62. doi:10.36469/jheor.2026.156118

Figure S1: Markov Traces for Each Treatment in (A) EA Population and (B) ENA Population

Table S1: Treatment C: Exclusion of Background Costs

Table S2: EA Results with 5-Year Pause

Table S3: ENA Results with 5-Year Pause

Table S4: EA Results with 20-Year Pause

Table S5: ENA Results with 20-Year Pause

Table S6: EA Results with 10-Year Pause and 1.5% Benefit Discount Rate

Table S7: ENA Results with 10-Year Pause and 1.5% Benefit Discount Rate

This supplementary material has been provided by the authors to give readers additional information about their work.

This is an open-access article distributed under the terms of the Creative Commons Attribution 4.0 International License (CCBY-4.0). View this license's legal deed at http://creativecommons.org/licenses/by/4.0 and legal code at http://creativecommons.org/licenses/by/4.0/legalcode for more information.

Figure S1. Markov Traces for Each Treatment for (**A**) EA Population and (**B**) ENA Population

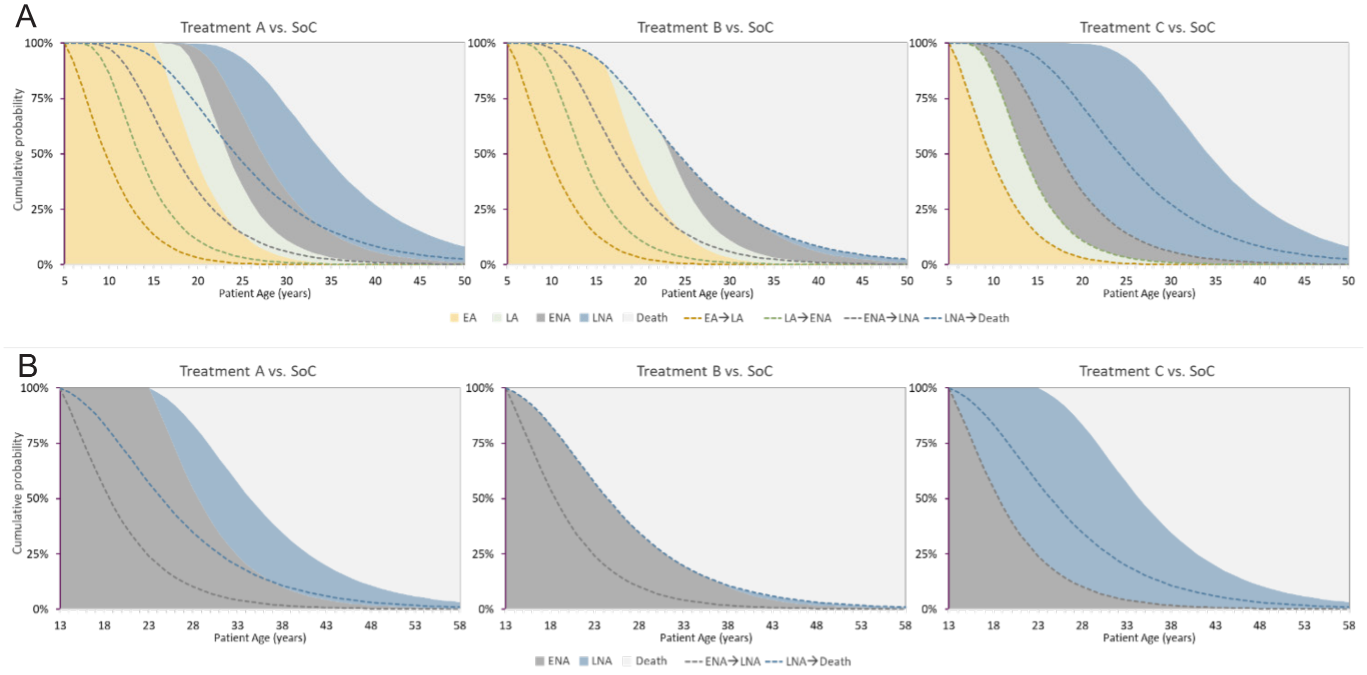

Abbreviations: EA, early ambulatory; ENA, early nonambulatory; LA, late ambulatory; LNA, late nonambulatory; SoC, standard of care.
EA (**A**) and ENA (**B**) populations with a 10-year pause. Dashed lines represent SoC risk curves; shaded areas represent the survival partition for treated patients. Treatment A delays nonfatal progression and mortality; Treatment B delays nonfatal progression only; Treatment C delays mortality only.

Table S1. Treatment C:[a] Exclusion of Background Costs

	**EA**	**ENA**
QALYs gained vs SoC	0.88	1.10
Incremental costs vs SoC, $	0	0
Total LY	20.15	16.55
Maximum annual treatment price, $		
$50 000/QALY	2179	3325
$100 000/QALY	4358	6650
$150 000/QALY	6537	9975
$200 000/QALY	8716	13 300

Abbreviations: EA, early ambulatory; ENA, early nonambulatory; LY, life-years; QALY, quality-adjusted life-year; SoC, standard of care.
Maximum annual treatment price represents the average across the full treatment time, which is different between the EA and ENA populations (20.15 and 16.55 discounted LY, respectively).
[a]Delays death only.

Table S2. EA Results with 5-Year Pause

	Treatment A	Treatment B	Treatment C
QALYs gained vs SoC	2.48	2.00	0.47
Incremental costs vs SoC, $	43 658	–72 046	117 524
Total LY	17.89	15.27	17.89
Maximum annual treatment price, $			
$50 000/QALY	4497	11 264	–5252
$100 000/QALY	11 435	17 811	–3934
$150 000/QALY	18 373	24 357	–2617
$200 000/QALY	25 310	30 904	–1299

Abbreviations: EA, early ambulatory; LY, life-years; QALY, quality-adjusted life-year; SoC, standard of care.
Maximum annual treatment price represents the average across the full treatment time, which is different between Treatments A and C (17.89 discounted LY) and Treatment B (15.27 discounted LY). Treatment A delays nonfatal progression and mortality; Treatment B delays nonfatal progression only; Treatment C delays mortality only.

Table S3. ENA Results with 5-Year Pause

	Treatment A	Treatment B	Treatment C
QALYs gained vs SoC	0.71	0.10	0.59
Incremental costs vs SoC, $	103 780	−36 792	147 325
Total LYs	13.72	10.44	13.72
Maximum annual treatment price, $			
$50 000/QALY	−4987	3996	−8584
$100 000/QALY	−2409	4467	−6431
$150 000/QALY	169	4938	−4277
$200 000/QALY	2746	5409	−2123

Abbreviations: ENA, early nonambulatory; LY, life-year; QALY, quality-adjusted life-year; SoC, standard of care.
Maximum annual treatment price represents the average across the full treatment time, which is different between Treatments A and C (13.72 discounted LY) and Treatment B (10.44 discounted LY). Treatment A delays nonfatal progression and mortality; Treatment B delays nonfatal progression only; Treatment C delays mortality only.

Table S4. EA Results with 20-Year Pause

	Treatment A	Treatment B	Treatment C
QALYs gained vs SoC	8.06	4.10	1.53
Incremental costs vs SoC, $	141 816	-122 514	381 776
Total LYs	23.78	15.27	23.78
Maximum annual treatment price, $			
$50 000/QALY	10 992	21 457	−12 835
$100 000/QALY	27 948	34 890	−9615
$150 000/QALY	44 903	48 324	−6395
$200 000/QALY	61 859	61 758	−3175

Abbreviations: EA, early ambulatory; LY, life-year; QALY, quality-adjusted life-year; SoC, standard of care.
Maximum annual treatment price represents the average across the full treatment time, which is different between Treatments A and C (23.78 discounted LYs) and Treatment B (15.27 discounted LYs). Treatment A delays nonfatal progression and mortality; Treatment B delays nonfatal progression only; Treatment C delays mortality only.

Table S5. ENA Results with 20-Year Pause

	Treatment A	Treatment B	Treatment C
QALYs gained vs SoC	2.30	0.13	1.92
Incremental costs vs SoC, $	337 124	−48 573	478 581
Total LYs	21.10	10.44	21.10
Maximum annual treatment price, $			
$50 000/QALY	−10 532	5276	−18 131
$100 000/QALY	−5088	5897	−13 582
$150 000/QALY	356	6519	−9033
$200 000/QALY	5800	7141	−4485

Abbreviations: ENA, early nonambulatory; LY, life-year; QALY, quality-adjusted life-year; SoC, standard of care.
Maximum annual treatment price represents the average across the full treatment time, which is different between Treatments A and C (21.10 discounted LYs) and Treatment B (10.44 discounted LYs). Treatment A delays nonfatal progression and mortality; Treatment B delays nonfatal progression only; Treatment C delays mortality only.

Table S6. EA Results with 10-Year Pause and 1.5% Benefit Discount Rate

	**Treatment A**	**Treatment B**	**Treatment C**
QALYs gained vs SoC	5.76	4.08	1.24
Incremental costs vs SoC, $	81 317	−109 694	218 901
Total LYs	24.68	17.78	24.68
Maximum annual treatment price, $			
$50 000/QALY	8373	17 645	−6354
$100 000/QALY	20 041	29 118	−3837
$150 000/QALY	31 710	40 592	−1320
$200 000/QALY	43 378	52 065	1197

Abbreviations: EA, early ambulatory; LY, life-year; QALY, quality-adjusted life-year; SoC, standard of care.
Maximum annual treatment price represents the average across the full treatment time, which is different between Treatments A and C (24.68 discounted LY) and Treatment B (17.78 discounted LY). Treatment A delays nonfatal progression and mortality; Treatment B delays nonfatal progression only; Treatment C delays mortality only.

Table S7. ENA Results with 10-Year Pause and 1.5% Benefit Discount Rate

	**Treatment A**	**Treatment B**	**Treatment C**
QALYs gained vs SoC	1.65	0.15	1.39
Incremental costs vs SoC, $	193 301	−47 347	274 407
Total LYs	19.48	11.74	19.48
Maximum annual treatment price, $			
$50 000/QALY	−5699	4668	−10 513
$100 000/QALY	−1474	5304	−6939
$150 000/QALY	2751	5940	−3365
$200 000/QALY	6975	6576	209

Abbreviations: ENA, early nonambulatory; LY, life-year; QALY, quality-adjusted life-year; SoC, standard of care.
Maximum annual treatment price represents the average across the full treatment time, which is different between Treatments A and C (19.48 discounted LY) and Treatment B (11.74 discounted LY). Treatment A delays nonfatal progression and mortality; Treatment B delays nonfatal progression only; Treatment C delays mortality only.
